# Our Journal

**Published:** 1850-03

**Authors:** 


### ARTICLE IV.

### OUR JOURNAL.

As we close the present volume of our work, we ask indulgence for any failure to render it what it ought to be, we may have made; while we shall enter upon a new year, with renewed encouragement, as we are almost daily receiving accessions to our list of subscribers, and as our contributors have become so numerous and so liberal in their aid, that we feel strong in that support, that renders our work valuable.—Among other original matter, of a highly interesting character, already on hand, we have a complete history of the Medical Profession in the United States, from the first settlement of the Colonies, down to the present time, by Prof. Davis; sixteen pages of which will be inserted in each number of the next volume. We promise our readers in advance, that they will be highly gratified by the perusal of this history; while they acquire what every physician should have i. e. a knowledge of the profession and its great men in their own country, and which is no where else to be found collated.

We shall extend our article headed Miscellaneous Medical Intelligence, so as to give all the current news of a professional character, that we may deem interesting to our readers.—We also, have made arrangements by which the mechanical execution of our printing shall be improved.

That we may be able to extend the usefulness of our work, and improve it still farther, we ask our friends to aid us in extending its circulation. E.

